# Hsa-miR-125b Therapeutic Role in Colon Cancer Is Dependent on the Mutation Status of the TP53 Gene

**DOI:** 10.3390/pharmaceutics13050664

**Published:** 2021-05-06

**Authors:** Diana Cenariu, Alina-Andreea Zimta, Raluca Munteanu, Anca Onaciu, Cristian Silviu Moldovan, Ancuta Jurj, Lajos Raduly, Alin Moldovan, Adrian Florea, Liviuta Budisan, Laura Ancuta Pop, Lorand Magdo, Mihai Tudor Albu, Rares Bogdan Tonea, Mihai-Stefan Muresan, Calin Ionescu, Bogdan Petrut, Rares Buiga, Alexandru Irimie, Diana Gulei, Ioana Berindan-Neagoe

**Affiliations:** 1 MEDFUTURE—Research Center for Advanced Medicine, “Iuliu-Hatieganu” University of Medicine and Pharmacy, Marinescu 23 Street/Louis Pasteur 4–6 Street, 400337 Cluj-Napoca, Romania; diana.cenariu@umfcluj.ro (D.C.); zimta.alina.andreea@gmail.com (A.-A.Z.); muresan.raluca.andrada@gmail.com (R.M.); ancaonaciu@gmail.com (A.O.); moldovan.cristian1994@gmail.com (C.S.M.); alin.moldovan@umfcluj.ro (A.M.); 2 Research Center for Functional Genomics, Biomedicine and Translational Medicine, “Iuliu Hatieganu” University of Medicine and Pharmacy, 23 Marinescu Street, 400337 Cluj-Napoca, Romania; ancajurj15@gmail.com (A.J.); raduly.lajos78@gmail.com (L.R.); liviutabudisan@gmail.com (L.B.); laura.ancuta.pop@gmail.com (L.A.P.); ioananeagoe29@gmail.com (I.B.-N.); 3 Department of Cell and Molecular Biology, Faculty of Medicine, “Iuliu Haţieganu” University of Medicine and Pharmacy, 6 Louis Pasteur St., 400349 Cluj-Napoca, Romania; aflorea@umfcluj.ro; 4 Faculty of Medicine, “Iuliu-Hatieganu” University of Medicine and Pharmacy, 8 Victor Babes Street, 400012 Cluj-Napoca, Romania; lorand.magdo@gmail.com (L.M.); albumihaitudor@gmail.com (M.T.A.); tonearares@gmail.com (R.B.T.); 5 5th Surgical Department, Municipal Hospital, 11 Tăbăcarilor Street, 400139 Cluj-Napoca, Romania; mihai.stefan.muresan@gmail.com (M.-S.M.); calin.ionescu@umfcluj.ro (C.I.); 6 Surgical and Gynecological Oncology Department, Prof. Dr. Ion Chiricuta” Oncology Institute, Republicii 34–36 Street, 400015 Cluj-Napoca, Romania; 7 Department of Surgery V, “Iuliu Hatieganu” University of Medicine and Pharmacy, 8 Victor Babes Street, 400012 Cluj-Napoca, Romania; 8 Department of Urology, “Prof. Dr. Ion Chiricuta” Oncology Institute, Republicii 34–36 Street, 400015 Cluj-Napoca, Romania; bogdan.petrut@gmail.com; 9 Department of Urology, “Iuliu-Hatieganu” University of Medicine and Pharmacy, 8 Victor Babes Street, 400012 Cluj-Napoca, Romania; 10 Department of Pathology, “Prof. Dr. Ion Chiricuta” Oncology Institute, Republicii 34–36 Street, 400015 Cluj-Napoca, Romania; raresbuiga@umfcluj.ro; 11 Department of Pathology, “Iuliu-Hatieganu” University of Medicine and Pharmacy, 8 Victor Babes Street, 400012 Cluj-Napoca, Romania; 12 11th Department of Surgical Oncology and Gynaecological Oncology, “Iuliu Hatieganu” University of Medicine and Pharmacy, 8 Victor Babes Street, 400012 Cluj-Napoca, Romania; airimie@umfcluj.ro; 13 Department of Surgery, The Oncology Institute “Prof. Dr. Ion Chiricuta”, 34–36 Republicii Street, 400015 Cluj-Napoca, Romania; 14 Department of Functional Genomics and Experimental Pathology, “Prof. Dr. Ion Chiricuta” Oncology Institute, 34–36 Republicii Street, 400015 Cluj-Napoca, Romania

**Keywords:** colon cancer, colorectal cancer, miR-125b, therapy, *TP53*, mutation

## Abstract

Colon cancer is the third most common cancer type worldwide and is highly dependent on DNA mutations that progressively appear and accumulate in the normal colon epithelium. Mutations in the *TP53* gene appear in approximately half of these patients and have significant implications in disease progression and response to therapy. miR-125b-5p is a controversial microRNA with a dual role in cancer that has been reported to target specifically *TP53* in colon adenocarcinomas. Our study investigated the differential therapeutic effect of miR-125b-5p replacement in colon cancer based on the *TP53* mutation status of colon cancer cell lines. In *TP53* mutated models, miR-125b-5p overexpression slows cancer cells’ malignant behavior by inhibiting the invasion/migration and colony formation capacity via direct downregulation of mutated *TP53*. In *TP53* wild type cells, the exogenous modulation of miR-125b-5p did not significantly affect the molecular and phenotypic profile. In conclusion, our data show that miR-125b-5p has an anti-cancer effect only in *TP53* mutated colon cancer cells, explaining partially the dual behavior of this microRNA in malignant pathologies.

## 1. Introduction

Colorectal cancer, including both cancer of the colon and cancer of the rectum, is the third most frequently diagnosed cancer type in developed countries, with a steady increase in incidence and mortality in the past few years. It mostly affects the male population and is heavily linked with lifestyle choices, such as high consumption of processed meat, smoking tobacco, alcohol consumption, and obesity. If discovered in early stages, the prognostic is positive, with almost full recovery after surgery; however, more than 25% of cases are discovered in late stages [1,2], when local affection of the lymph nodes is present as well as possible distant metastasis. In this case, the prognostic is reserved and the primary treatment option is chemotherapy or targeted therapies with a limited increase in patients’ survival [3].

MicroRNAs (miRNAs) are short non-coding RNAs (ncRNAs), with 19–25 nucleotides in length that can impair protein translation by binding through a seed region of 3–5 nucleotides of the messenger RNA (mRNA) [4,5,6]. miRNAs are associated with a broad spectrum of pathological states, including cancer [2,7,8]. Their ability to target multiple genes at the same time and also their aberrant expression in malignant pathologies, make these small non-coding sequences attractive therapeutic targets in oncology [9,10], including colon cancer. Despite intensive preclinical research, a limited number of studies involving miRNA-based therapeutics in cancer have shift to the clinical testing stage [11]. Among the reasons, could be the fact that numerous studies with miRNAs in cancer are analyzing the direct correlation of expression between a specific miRNA and a targeted mRNA, frequently disregarding the mutational status of the targeted gene and its role in the context of modified genetics. Considering that a tumor suppressor gene can acquire mutations with oncogenic roles outside of the miRNA binding sites (e.g., *TP53*), the tumor suppressor value of a miRNA can switch to oncogenic and vice versa in respect to the genetic integrity of the target genes. Moreover, the large plethora of targeted genes of a specific miRNA and the signaling connection between them in different cellular context is most of the time disregarded in favor of a restricted pool of targeted genes that do not mirror the whole spectrum of pathological changes from the cellular level. This is the case of miR-125b-5p that has a controversial status in cancer, including colon cancer [12]. The discrepancies between studies showing its oncogenic or tumor suppressor role could be related to the specific function of the targeted genes in respect to their mutational status. Previous studies have shown that high expression of miR-125b in colon cancer is associated with negative prognosis in the context of a direct inhibition of *TP53* network [13]. Other studies showed that miR-125b suppresses colorectal cancer cells invasion and proliferation through inhibition of PDZ-binding motif (*TAZ*) [14].

To tackle these contradictory results, we decided to investigate the role of miR-125b in colon cancer in reliance of the mutation status of *TP53* target gene. Genetic alteration of *TP53* is present in approximately half of all colorectal cancers. While mutations in *TP53* are not necessarily essential for patients undergoing curable resection, they are decisive for those treated with chemotherapy with a negative impact upon survival [15]. For example, in vitro studies showed that the response to 5-fluorouracil (5-FU)-based chemotherapy is complete only in colon cancer models with wild-type *TP53*; however, retrospective studies or incorporation of *TP53* mutation status in clinical studies could demonstrate the translational value of this dependency [15].

Considering the opposing roles of *TP53* in colon cancer based on the mutational status, tumor suppressor in the case of wild-type forms and oncogenic for some mutant forms, and also the significant percent of patients with *TP53* mutation, we employed two different cellular models of colon cancer to investigate the therapeutic role of miR-125b: wild-type and mutant *TP53* colon cancer cells. Although in our study miR-125b presented a uniform downregulated level in both patient tumor samples and *TP53* wild-type and mutant colon cancer cell lines, the therapeutic role of miRNA replacement was observed only in the case of mutant *TP53* study models, mutation identified as pathogenic. Therefore, replacement of miR-125b could be an advantageous strategy for those patients that present oncogenic driven mutation in the *TP53* genes, patients that are also the most susceptible to lymphatic invasion, uncomplete therapeutic response and poor survival.

This study is meant to demonstrate, besides the specific activity of miR-125b in colon cancer, that experimental therapeutics with miRNAs should involve a close look at the mutational status of the target genes, and how these genetic abnormalities are changing the spectrum of the other targeted transcripts, even in the case of a uniform dysregulation of the miRNA in large patient cohorts.

## 2. Methods and Materials

### 2.1. Tissue Samples Analysis of miR-125b Expression

miR-125b expression was analyzed in three cancer types from tumor and normal adjacent tissue samples from the “Prof. Dr. Ion Chiricuta” Institute of Oncology, Cluj-Napoca, Romania. The included patients were diagnosed with double positive breast cancer (21 normal adjacent tissue samples and 44 tumor tissue samples), bladder cancer (39 normal adjacent tissue samples and 37 tumor tissue samples), and colon cancer (23 normal adjacent tissue samples and 25 tumor tissue samples). The tissue samples were taken from the surgically removed tissue during routine surgery and did not pose additional risk for the patients. All patients signed an informed consent for inclusion in the study that was approved by the Ethical Committee of the Institute. The protocol for use of human samples in the current experiment was approved by the Ethical Committee of the Institute and by the Ethical Committee of the Iuliu Hatieganu University of Medicine and Pharmacy, Cluj-Napoca, Romania.

### 2.2. TCGA Analysis of miRNA/mRNA Expression

The validation of the miRNA expression was made using the RNASeq counts data available at The Tumor Genome Atlas (TCGA) database retrieved from University of California Santa Cruz Cancer Browser. RNASeq counts data for miR-125b expression was organized for each of the pathologies of interest (breast cancer, bladder cancer and colon cancer) and the samples separated in normal and tumoral tissue, depending on the TCGA sample code. The counts (normal tissue versus tumor tissue) were exported in GraphPad Prism (© 2021 GraphPad Software, San Diego, CA, USA) where the Mann–Whitney U test was applied to compare the two groups. For a more complete comparison of miR-125b expression across different malignant pathologies (comparison between normal tissue and tumor tissue), the dbDEMC (database of Differentially Expressed MiRNAs in human Cancers) was used. This database contains integrated data from Gene Expression Omnibus (GEO) and The Cancer Genome Atlas (TCGA), as well as manually curated experimental reports on 2224 miRNAs with differential expression in cancer [16].

For evaluation of gene expression between normal, *TP53* wild-type colon cancer and *TP53* mutant colon cancer samples, we used the UALCAN database (http://ualcan.path.uab.edu/cgi-bin/ualcan-res.pl accessed on 15 October 2020), which is based on gene expression extracted from TCGA RNA Seq data correlated with the clinical data. In this database, the name of each gene was introduced first, then its expression based on the *TP53* mutation status was chosen. The results were presented as a box plot graph.

### 2.3. Cell Culture and Spheroid Formation

Two colon cancer cell lines were used for the study: HCT-116 (ATCC^®^ CCL-247™, Manassas, VA, USA) and DLD-1 (ATCC^®^ CCL-221™, Manassas, VA, USA) and one normal fibroblast cell line: CCD-18Co (ATCC^®^ CRL-1459™, Manassas, VA, USA). The cancer cell lines are originating from colorectal patients, although the transformed cells are from the colon biopsy. HCT-116 cell line was cultured in Mc Coy’s media (Merck KGaA, Darmstadt, Germany) supplemented with 10% fetal bovine serum (FBS), DLD-1 cell line was cultured in RPMI media (Merck KGaA, Darmstadt, Germany) supplemented with 10% FBS, while CCD-18Co cell line was cultured in MEM media (Merck KGaA, Darmstadt, Germany) supplemented with 10% FBS according to the provider protocol. All cell lines were passaged 2–3 times a week and were incubated at 37 °C with 5% CO_2_. The 3D spheroids of DLD-1 were obtained through the hanging drops technique as previously exemplified [17].

### 2.4. Cell Transfection

HCT-116 and DLD-1 cell lines were transfected with miRNA mimic and inhibitor using Lipofectamine 2000 (Invitrogen), diluted in OptiMEM media (Gibco, Thermo Fisher Scientific, Carlsbad, CA, USA) following the producer instructions. The control cell groups were transfected with empty Lipofectamine 2000 in the same concentration. For hsa-miR-125b mimic (Invitrogen, Thermo Fisher Scientific, Carlsbad, CA, USA) and hsa-miR-125b inhibitor (Invitrogen, Thermo Fisher Scientific, Carlsbad, CA, USA) transfection it was used 100 nM of miRNA mimic and inhibitor (based on literature data), as follows: the oligonucleotides were diluted in OptiMEM (Merck KGaA, Darmstadt, Germany) and then incubated for 5 min at RT with Lipofectamine 2000 (Thermo Fisher Scientific, Waltham, MA, USA) diluted also in OptiMEM. After 6 h, the OptiMEM media was removed and replaced with the corresponding culture media, and then 48 h after transfection, the cells were ready for functional and molecular assays.

### 2.5. Flow Cytometry Analysis on Cell Cycle and Apoptosis/Necrosis

The miRNA transfected and the control cell suspension was washed three times with PBS 1X (Merck KGaA, Darmstadt, Germany) and fixed with pre-chilled 70% ethanol for 50 min for the analysis of cell cycle progression. Afterward, the cells were rewashed with PBS 1X and treated with RNase buffer (40 µg/mL RNase and 0.2% BSA) (Thermo Fisher Scientific, Waltham, MA, USA) for 15 min, at RT. At the end of this time, the propidium iodine (Thermo Fisher Scientific, Waltham, MA, USA) was added in dark conditions at a final concentration of 10 µg/mL in 500μL buffer and incubated at RT for another 30 min.

For apoptosis/necrosis ratio analysis at flow cytometry, the miRNA transfected, and control cells were trypsinized, resuspended in complete media and washed 3 times with PBS 1X. After the washing step the cells were centrifuged at 1200 rpm for 5 min and mixed with Annexin V Binding buffer (Invitrogen, Thermo Fisher Scientific, Carlsbad, CA, USA). The cells were then stained with 5 μL Annexin V (Thermo Fisher Scientific, Waltham, MA, USA) and 5 μL propidium iodide (Merck KGaA, Darmstadt, Germany) for 15 min.

The samples were read at the flow cytometer BD FACS Canto II instrument (BD, San Jose, CA, USA). The initial gaiting was fixed based on the control for each cell line. The data was analyzed with FACS Diva version 6.0 software (BD, San Jose, CA, USA).

### 2.6. Wound Healing Assay

The miRNA transfected and control cells were plated into a 24-well plate designed with a silicon insert (Ibidi, GmbH, Blossom Biotechnologies Inc., Gräfelfing, Germany). The cells were plated at 40,000 cells/well and left to adhere for 24 h. After 24 h, the inserts were removed, and pictures were taken at 0 h, 12 h, 24 h, and 48 h. The covering of the scratch area was analyzed with the help of MiToBo plug-in in Image J software.

### 2.7. Colony Assay

After 48 h from transfection the transfected and control cells were trypsinized and diluted until reaching 1000 cells/mL, then distributed in triplicates in a 6 well plate, 500 cells/well. The colonies were left to form for three weeks, then they were fixated with 80% methanol, stained with 0.5% crystal violet, and then washed until only the colonies remained stained. After the plate was dry, pictures of each well were taken with the help of a Canon camera. The number of colonies and their respective size, as well as the image processing was done with Image J software.

### 2.8. Extracellular Matrix 3D Invasion Assay

The protocol for extracellular matrix 3D invasion assay was adapted from the 3D invasion assay by Berens E.B. et al. [18]. After the spheroids from transfected and control cells were formed through the hanging drop technique as previously reminded, they were recovered and immersed in a 1/1 Geltrex stock solution (Thermo Fisher Scientific, Waltham, MA, USA) and 0.25% Collagen type I solution (Thermo Fisher Scientific, Waltham, MA, USA). Photos were taken at 0 h and 48 h. The exterior limits of the invasion were marked and measured with the help of Image J software.

### 2.9. Confocal Fluorescence Microscopy

HCT-116 and DLD-1 cell suspensions were pipetted in chamber slide and left to adhere. After 24 h, the cells were transfected with empty or miR-125b mimic/inhibitor-containing Lipofectamine 2000 as previously stated; 48 h post-transfection the cells were triple-strained for nucleus, mitochondria, and cytoskeleton based on a coloration protocol previously developed in our laboratory [19]. First, the cells were incubated for 4 h with MitoTracker (Thermo Fisher Scientific, Waltham, MA, USA), then fixed with 4% paraformaldehyde and treated with Triton X detergent for 15 min. Following detergent treatment and washing three times with PBS, the actin filaments were stained with Phalloidin-FITC (Cytoskeleton, lnc, Denver, CO, USA) for 15 min, washed again with PBS and finally stained with DAPI (Thermo Fisher Scientific, Waltham, MA, USA) for the nucleus for 1 min. The pictures were taken with the help of Olympus FLUOVIEW FV1200 laser scanning fluorescence confocal microscope (Olympus, Tokyo, Japan).

### 2.10. RNA Isolation and RT-qPCR for miRNA and mRNA Expression

Transfected and control cells were resuspended in 800 µL of TRI Reagent (Sigma Aldrich, Saint Louis, MO, USA) and immediately froze in liquid nitrogen. The RNA was extracted from TRI Reagent based on the chloroform-ethanol extraction method and quantified with NanoDrop-1100. For reverse transcription of miRNA sequences, we used the TaqMan MicroRNA Reverse Transcription Kit (Applied Biosystems, Foster City, CA, US), whereas, for reverse transcription of mRNA, we used High Capacity cDNA Reverse Transcription Kit (Applied Biosystems, Foster City, CA, USA). The RT-PCR reaction was done with SsoAdvanced Universal Probes Supermix (Bio-Rad, Hercules, CA, USA) for miRNA evaluation and SYB Select Master Mix (Bio-Rad, Hercules, CA, USA) for the expression of protein-coding genes. The miRNA expression and mRNA expression were done with the ViiA™ 7 System (Thermo Fischer Scientific, Waltham, MA, USA).

### 2.11. DNA Isolation and NGS Analysis

DNA from HCT-116 and DLD-1 colon cancer cell lines was extracted using the Purelink Genomic DNA minikit (Thermofisher Scientific, Waltham, MA, USA) following the manufacturer instruction. 20 ng of DNA were used for sequencing using the Ion AmpliSeq Cancer Hotspot Panel v2 (ThermoFischer Scientific, Waltham, MA, USA) and the Ion AmpliSeq Library 2.0 kit (Thermofisher Scientific, Waltham, MA, USA). The Ion AmpliSeq Cancer Hotspot Panel v2 consists of primers for hotspot evaluation in the following genes: *ABL1, AKT1,ALK, APC, ATM, BRAF, CDH1, CDK2A, CSF1R, CTNNB1, EGFR, ERBB2, ERBB4, EZH2, FBXW7, FGFR1, FGFR2, FGFR3, FLT3, GNA11, GNAQ, GNAS, HNF1A, IDH1, IDH2, JAK2, JAK3, KDR, KIT, KRAS, MET, MLH1, MPL, NOTCH1, NPM1, NRAS, PDGFRA, PIK3CA, PTEN, PTPN11, RB1, RET, SMAD4, SMARB1, SMO, STK11, TP53,* and *VHL*. After library preparation the samples were purified using the AMpure XP Beads (Beckman Coulter, Inc., Brea, CA, USA). The purified libraries were quantified using the fluorometer Qubit 2.0 (Sigma Aldrich, Saint Louis, MO, USA) and the Qubit HS DNA kit (Sigma Aldrich, Saint Louis, MO, USA). For template synthesis, libraries were diluted to 100 pM and multiplex 2 libraries on an Ion 316 Chip (ThermoFischer Scientific, Waltham, MA, USA). The sequencing process was performed on the Ion Torrent PGM Machine (ThermoFischer Scientific, Waltham, MA, USA) using the Ion PGM HI-Q Sequencing 200 kit (ThermoFischer Scientific, Waltham, MA, USA). The data obtained after sequencing were analyzed using the Torrent Suit 5.6 and Ion Reporter 5.6 software for data trimming, alignment and variant calling. The obtained variants were filtered using the following conditions: *p* value ≤ 0.05, coverage ≥ 500.

### 2.12. Statistical Analysis

All results were evaluated statistically in GraphPad Prism software, with the help of two-tailed *t*-test and nonparametric tests. Statistical significance was considered a *p*-value greater than 0.05. Mann–Whitney U test was applied in the case of TCGA and local cohorts screening for miR-125b and ANOVA test to assess the difference between the three groups in the case of the scratch assay. The rest of the experiments were analyzed through paired experimental design, using paired t test.

## 3. Results

### 3.1. miR-125b Has a Heterogenous Expression Profile Across Cancer Types

**Figure 1:**
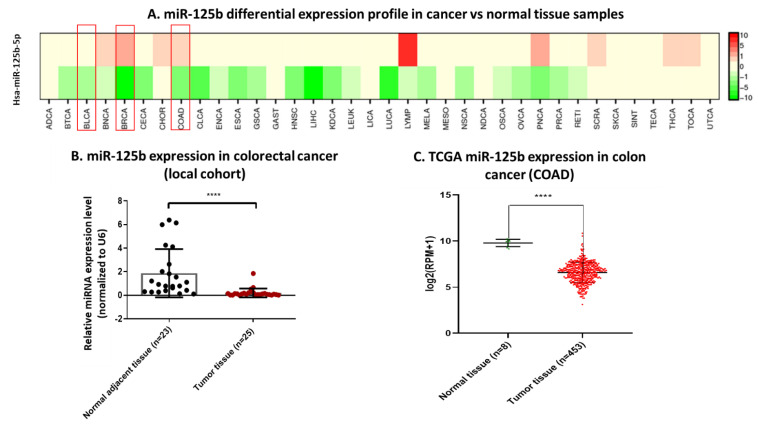
Screening of miR-125b expression in cancer, with a focus on colon malignancies. (**A**) miR-125b-5p expression screening across cancer types; data retrieved from dbDEMC (database of Differentially Expressed MiRNAs in human Cancers). Within the red squares there are the three in depth analyzed cancer types: breast cancer (BRCA), bladder cancer (BLCA), and colon cancer (COAD). (**B**) RT-qPCR results of miR-125b-5p expression in colon cancer tissue samples (*n* = 25) versus normal adjacent tissue samples (*n* = 23) (data presented as mean ± S.D.; **** *p* < 0.0001, two-tailed Mann–Whitney test). Experiments including the local cohorts of patients were performed in duplicate for each sample and miR-125b-5p relative expression was expressed by fold-change and normalized to U6 snRNA (**C**) Validation of miR-125b-5p expression (RNASeq count) on COAD cohort from TCGA database in tumor tissue (*n* = 453) compared to normal ones (*n* = 8) (data presented as mean ± S.D.; **** *p* < 0.0001, two-tailed Mann–Whitney test).

Analysis of miR-125b expression in cancer tissue compared to normal samples from combined data retrieved from TCGA, GEO and literature (dbDEMC 2.0 database) showed that this miRNA is heterogeneous in expression between cancer types and also within the same malignancy (Figure 1A). Confirmation of miR-125b expression was done on three separate cancer types: double positive breast cancer, bladder cancer (Figure S1), and colon cancer (Figure 1B,C) on local cohorts and TCGA data. Analysis of miR-125b expression in tissue samples from local cohorts showed this miRNA is downregulated in all the analyzed cancer types (double positive breast cancer—Figure S1A, bladder cancer—Figure S1B, and colon cancer—Figure 1B) and in TCGA cohorts for the same malignancies (double positive breast cancer—Figure S1C, bladder cancer—Figure S1D, and colon cancer—Figure 1C).

Specific for breast cancer, the RT-qPCR results done on the local cohort presented some outliers (*n* = 3) in tumor tissue with upregulated expression compared to normal adjacent tissue, but with an overall statistically significant downregulated profile (Figure S1A). These results were validated on the RNASeq counting data from the TCGA breast cancer cohort (BRCA) containing 104 normal tissue samples and 1098 tumor tissue samples (Figure S1C). In bladder cancer, the analysis of local cohort revealed that the expression of this miRNA is heterogenous across tumor and normal adjacent samples. However, the median expression of miR-125b is down-regulated in bladder cancer (Figure S1B), results confirmed on the analysis done on TCGA RNASeq data from the bladder cancer cohort (BLCA) on the same miRNA (Figure S1D).

The downregulation of miR-125b in colon cancer shows uniformity in expression in the entire group of tumor tissue samples from the local cohort compared to adjacent normal tissue associated with the highest statistical significance from all three cancer types: **** *p* < 0.0001 (Figure 1B). The marked downregulation was also mirrored in TCGA data from the colon cancer cohort (COAD) (Figure 1C). However, the literature data combined with TCGA data showed contradictory reports on the expression of miR-125b in colon cancer, meaning that both overexpression and under expression of this miRNA were reported (Figure 1A). Due to the contradictory results within the literature regarding the tumor suppressor or oncogenic function of miR-125b in colon/colorectal malignancies we decided to profundity the mechanisms and associated phenotypes related to TP53 mutational status (a gene with high mutation frequency in colon cancer) and miRNA mediated modulation, as this gene was identified with strong evidence as a direct target of miR-125b (according to the data retrieved from mirTargetLink database: https://ccb-web.cs.uni-saarland.de/mirtargetlink/ accessed on 15 October 2020, data not presented).

### 3.2. Establishment of the Functional In Vitro Model for Investigation of mir-125b Role in Connection to TP53 Mutation Status in Colon Cancer

**Figure 2:**
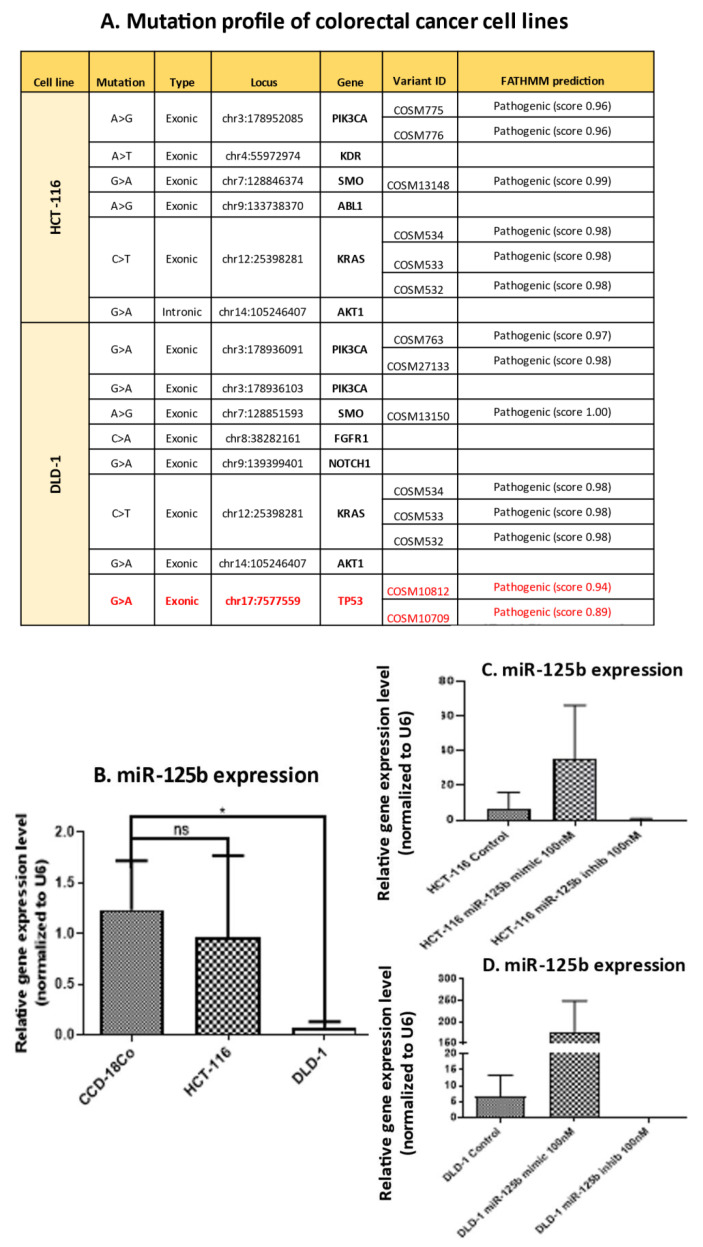
Establishment of functional in vitro model for miR-125b investigation in colon cancer. (**A**) Next-generation sequencing cancer panel results on HCT-116 and DLD-1 colon cancer cell lines. (**B**) RT-qPCR analysis for miR-125b-5p expression in colon cancer cell lines, HCT-116 and DLD-1, compared to normal cell line, CCD-18Co (normal colon fibroblasts) (data presented as mean ± S.D.; * *p* = 0.0161 for DLD-1, independent *t*-test compared to CCD-18Co). Experiments were performed in biological duplicates for each cell line and miR-125-5p relative expression was expressed by fold-change and normalized to U6 snRNA. (**C**,**D**). Validation of miR-125b mimic (100 nM) and inhibitor (100nM) transfection in HCT-116 cell line (**C**) and DLD-1 cell line (**D**) compared to control cell treated with empty Lipofectamine 2000 (data presented as mean ± S.D.). Experiments were performed in biological duplicates for each cell line and miR-125-5p relative expression was expressed by fold-change and normalized to U6 snRNA.

To establish the functional in vitro model for the study, two colon cancer cell lines, HCT-116 and DLD-1, were sequenced to investigate the mutation status of the *TP53* gene. As shown in Figure 2A, HCT-116 has fewer mutated genes in comparison with DLD-1. Most mutations present in both cell lines are located in exonic regions. *PIK3A* is mutated in both cell lines, with one A > G mutations in HCT-116 and two G > A SNPs in DLD-1 cell line. *KRAS*, a frequently mutated gene in colon cancer was found mutated in both cell lines. One of the most significant differences between the two cell lines is the presence of a G > A SNP in *TP53* gene, in an exonic region, in DLD-1 cell line. Although a rare mutation, as shown in the results mentioned above, this gene may cause a clear phenotype distinction between the two cell lines.

Furthermore, miR-125b expression was investigated in the two colon cancer cell lines compared to a normal cell line with the same origin, CCD-18Co. miR-125b is significantly down-regulated in DLD-1 cell line, which is *TP53* mutated when compared to the CCD-18Co cell line (Figure 2B). In HCT-116 cell line, which is TP53 wild type, miR-125b is slightly down-regulated compared to CCD-18Co, but without statistical significance (Figure 2B). Transfection of the two colon cancer cell lines with miR-125b mimic and inhibitor respectively showed the two cell lines’ capacity to accumulate the exogenous nucleotides and the complete inhibition of the minimal levels of miR-125b present before transfection (Figure 2C for HCT-116, and Figure 2D for DLD-1).

### 3.3. miR-125b Replacement Effect on Cell Cycle Progression and Cell Viability in Colon Cancer Cells Dependent on TP53 Status

**Figure 3:**
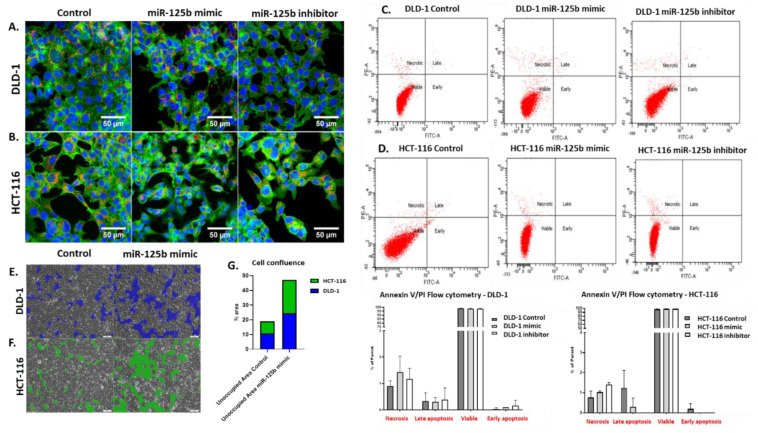
miR-125b modulation effects on cell viability. (**A**,**B**) Fluorescence staining of (**A**) DLD-1 and (**B**) HCT-116 marked for nucleus (blue), mitochondria (red) and cytoskeleton (green) used for detection via confocal microscopy of morphological changes upon miR-125b treatment with mimic sequences (100nM) and inhibitor sequences (100 nM) compared to control. The original picture contrast and luminosity were increased by 20%. (**C**,**D**) Flow cytometry analysis for detection of early and late apoptotic events, as well as necrosis for (**C**) DLD-1 and (**D**) HCT-116 treated with miR-125b mimic (100 nM) or inhibitor (100 nM) compared to control. Experiments were performed in biological triplicates for each cell line and each treatment scheme (data presented as mean ± S.D.; all the compared groups had a non-significant *p* value: *p* > 0.05, independent *t*-test compared to HCT-116 control). (**E**,**F**) Images of (**E**) DLD-1 and (**F**) HCT-116 cell confluence at 48 h after miR-125b mimic transfection (100 nM) and (**G**) analysis of occupied area. The pictures were taken with an inverse optical microscope at 4× magnification and processed with Image J software.

To analyze the morphological changes upon miR-125b replacement or inhibition, the treated cells were observed under triple coloration for the nucleus, mitochondria, and cytoskeleton with fluorescence dyes (Figure 3A,B). DLD-1 cell line transfected with miR-125b mimic shows increased cytoskeleton disruption and low apoptosis (Figure 3A). Lower levels of cytoskeleton disruption were also observed in the case of miR-125b inhibitor (Figure 3A). No significant changes were observed in terms of mitochondrial density (Figure 3A). In the case of HCT-116, the cells present significant levels of cellular stress, as shown by cytoskeletal disruption, initiation of apoptosis, increased gaps between cells and also decreased number of mitochondria (Figure 3B). In miR-125b inhibitor case, the cells have an enlarged nucleus, but overall, the morphology did not differ significantly between the control and inhibitor treated HCT-116 cells (Figure 3B).

The flow cytometry analysis of cellular population after miR-125b replacement and inhibition showed no statistically significant difference in the level of apoptotic/necrotic cells (Figure 3C,D). Although some trends can be observed, such as elevated levels of necrosis of both mimic and inhibitor treatment in HCT-116 and DLD-1, these can only be attributed to random events or non-specific activity of a foreign RNA transfection in a cell.

To better observe what happens upon transfection (and eliminate the processing steps of the cells before flow cytometry analysis where a significant part of the late apoptotic/necrotic cells can be lost due to centrifugation), pictures of the transfected cells were taken at 48 h after miR-125b mimic addition in comparison with control, directly into the transfection wells (Figure 3E,F). As can be observed, both cell lines treated with mir-125b mimic are affected in terms of confluency in comparison with the corresponding control. Specifically, the gaps between the adherent cells are increased, an event caused in most cases by cell death and decreased proliferation. No significant differences were observed between treated cell lines, with a slight increase in percentage of unoccupied area (approximately 1%) in the case of HCT-116 treated with miR-125b mimic compared to DLD-1 subjected to the same experimental (Figure 3G).

**Figure 4:**
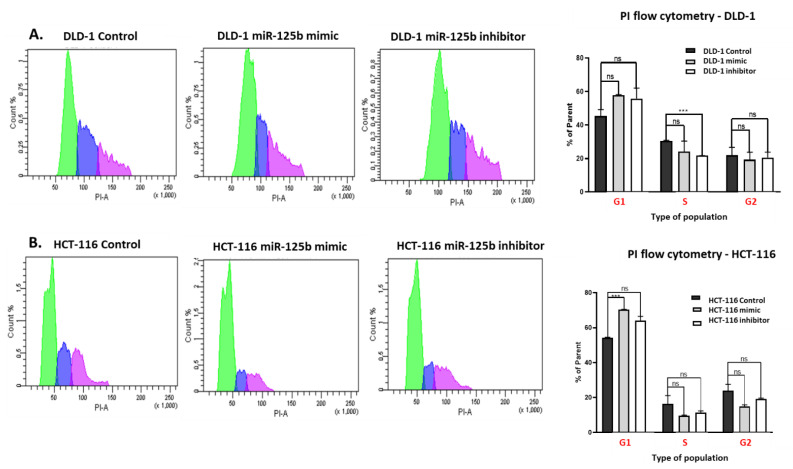
miR-125b modulation effects on cell cycle progression. (**A**,**B**) Flow cytometry analysis for detection of cell cycle progression for (**A**) DLD-1 and (**B**) HCT-116 treated with miR-125b mimic (100 nM) or inhibitor (100 nM) compared to control. Experiments were performed in biological triplicates for each cell line and each treatment scheme (data presented as mean ± S.D., *** *p* = 0.000542 for DLD-1 <phase S> treated with 125b inhibitor independent *t*-test compared to DLD-1 control; *** *p* = 0.000240 for HCT-116 <phase G1> treated with 125b mimic independent *t*-test compared to HCT-116 control). The colors present in the flow cytometry graphs for cell cycle analysis are representative for each phase of the cell cycle: green—G1 phase, blue—S phase, pink—G2 phase.

In cell cycle analysis, miR-125b mimic or inhibitor has no significant effect upon DLD-1 cycle progression, where the modifications of a cell population from G1, S, and G2 phase are similar between the opposite treatments to control (Figure 4A). miR-125b mimic administration in HCT-116 cell line significantly increased the number of cells situated in the G1 phase (Figure 4B), which is generally associated with an impairment in cell cycle progression. Concomitant with this increase, the same treatment pattern has determined the decrease of cells situated in the S phase of the cell cycle, although not statistically significant (Figure 4B). However, a similar pattern, but not so pronounced, can also be observed in case of miR-125b inhibitor treatment (Figure 4B), which suggests the concomitant involvement of a non-related miR-125b modulation factor (e.g., cellular stress due to the addition of an exogenous cargo).

### 3.4. miR-125b Exogenous Overexpression Inhibits the Invasion/Migration and Self-Renewal Capacity of TP53 Mutant Colon Cancer Cells

**Figure 5:**
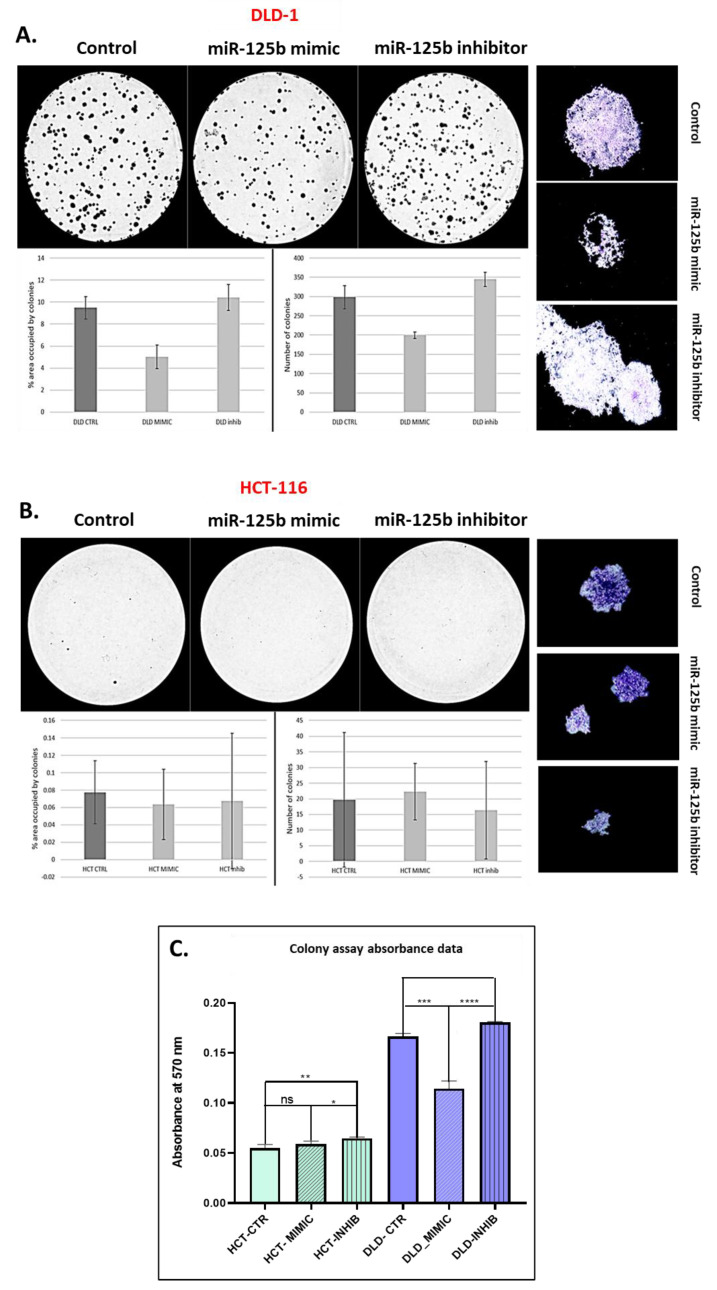
The effects of miR-125b replacement/inhibition on colon cancer cell self-renewal capacity. (**A**) Colony assay for DLD-1 cell line: top—colony assay pictures Control, miR-125b mimic (100nM) and miR-125b inhibitor (100nM) groups; bottom—graphical representation of the number of colonies and covered area (data presented as mean ± S.D., ns independent *t*-test compared to control); left—pictures of the individual colonies taken with an inverse optical microscope at 4× magnification. Experiments were performed in biological duplicates for each cell line and each treatment scheme (**B**) Colony assay for HCT-116 cell line: top—colony assay pictures Control, miR-125b mimic (100 nM) and miR-125b inhibitor (100 nM) groups; bottom—graphical representation of the number of colonies and covered area (data presented as mean ± S.D., ns independent *t*-test compared to control); left—pictures of the individual colonies taken with an inverse optical microscope at 4× magnification. Experiments were performed in biological duplicates for each cell line and each treatment scheme (**C**) Graphical representation of the absorbance values and difference between HCT-116/DLD-1 Control, miR-125b mimic (100 nM), and miR-125b inhibitor (100 nM) groups. Experiments were performed in biological duplicates for each cell line and each treatment scheme (data presented as mean ± S.D., ** *p* = 0.0024 for HCT-116 treated with miR-125b inhibitor independent *t*-test compared to HCT-116 control; * *p* = 0.0130 for HCT-116 treated with miR-125b mimic independent *t*-test compared to HCT-116 treated with miR-125b inhibitor control; *** *p* = 0.0001 for DLD-1 treated with miR-125b mimic independent *t*-test compared to DLD-1 control; *** *p* = 0.0014 for DLD-1 treated with miR-125b inhibitor independent *t*-test compared to DLD-1 control; **** *p* < 0.0001 for DLD-1 treated with miR-125b mimic independent *t*-test compared to DLD-1 treated with miR-125b inhibitor). The absorbance was measured after dilution of crystal violet colored colonies and is attributed to the number of colonies formed.

The exogenous overexpression of miR-125b has different effects on the self-renewal capacity of the two analyzed cell lines, DLD-1 and HCT-116, as proven by the colony assay results (Figure 5A–C). For the case of DLD-1 cell line, which is *TP53* mutant, the numbers of colonies and covered area by the colonies is significantly decreased after miR-125b replacement, in comparison with the control cells, whereas in the case of miR-125b inhibited cells, the number of colonies and covered area were increased (opposite effect) (Figure 5A). Moreover, the aspect of the colonies is modified compared to the control group: for the miR-125b mimic, the colonies are smaller without a uniform aspect, while for miR-125b inhibited cells, the colonies are larger and began to unify very rapidly (Figure 5A). For the case of HCT-116, under the same protocol conditions as in the case of DLD-1, the cell line showed a minimum capacity of forming colony units, regardless of the experimental conditions (control, miR-125b mimic, or miR-125b inhibitor). Analysis of covered area, number of colonies and colony aspect was relatively uniform between the experimental conditions, thus showing non-significant difference between the self-renewal capacity (Figure 5B). However, upon reading the absorbance of diluted crystal violet, there was a slight difference between control and inhibitor groups and between mimic and inhibitor ones (Figure 5C). In the case of crystal violet absorbance for DLD-1 colonies there was a significant difference between control and mimic (*p* = 0.0001), control and inhibitor (*p* = 0.0014) and mimic and inhibitor groups (*p* < 0.0001) (Figure 4C).

**Figure 6:**
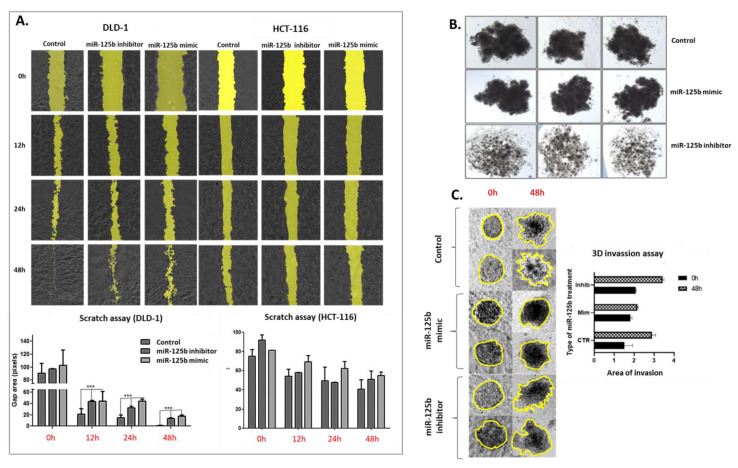
The effects of miR-125b replacement/inhibition on colon cancer cell invasion/migration. (**A**) Wound healing assay results of DLD-1 and HCT-116 cells control or treated with miR-125b mimic/inhibitor (100 nM): top—pictures of scratch closing through 48 h (0 h, 12 h, 24 h, and 48 h) processed with the help of Image J software; bottom—graphical representation of the gap site for the two cell lines in each of the experimental conditions at 0 h, 12 h, 24 h, and 48 h timepoints. Experiments were performed in biological duplicates for each cell line and each treatment scheme (data presented as mean ± S.D., *** *p* < 0.001 for DLD-1 at 12 h ANOVA test comparing control versus miR-125b mimic versus miR-125b inhibitor; *** *p* < 0.001 for DLD-1 at 24 h ANOVA test comparing control versus miR-125b mimic versus miR-125b inhibitor; *** *p* < 0.001 for DLD-1 at 48 h ANOVA test comparing control versus miR-125b mimic versus miR-125b inhibitor. (**B**) Aspect of spheroids of DLD-1 cell treated with miR-125b mimic (100 nM) or miR-125b inhibitor (100 nM). The representative pictures were taken with an inverse optical microscope at 4× magnification. (**C**) Pictures and graphical representation of the 3D invasion assay of the extracellular matrix of DLD-1 control, miR-125b mimic (100 nM) and miR-125b inhibitor (100 nM) spheroids. Area of spheroid is indicated by a yellow line. The graph contains the progression of the spheroid area. Experiments were performed in biological duplicates for each cell line and each treatment scheme (data presented as mean ± S.D.).

The wound-healing assay results mirror the ones obtained in colony assay, thus proving a dual effect of miR-125b in self-renewal and invasion (Figure 6A). Specifically, the generated scratch closed the fastest in DLD-1 cells treated with miR-125b mimic compared to control and miR-125b inhibitor treated cells (Figure 6A). When analyzing the difference in gap size between 0 h 12 h, 24 h, and 48 h, the miR-125b mimic group always had the largest gap with statistical significance than control for each time point. However, there was a difference in gap size also between control DLD-1 and inhibitor treated DLD-1 cells, with a larger gap size for the case of the inhibitor treated ones. This difference is demonstrating that miRNAs, including miR-125b, are hard to categorize as truly tumor suppressor or oncogenic, due to their extensive profile of targeted genes (83 targeted genes validated by strong experimental support according to data retrieved from mirTargetLink database: https://ccb-web.cs.uni-saarland.de/mirtargetlink/ accessed on 15 October 2020, data not presented). Most of the time, in the case of a tumor suppressor miRNAs, this profile is not exclusively formed of genes with oncogenic roles that are expressed upon exogenous miRNA inhibition or suppressed upon miRNA replacement. These intercalating pathways can therefore compensate in the case of specific mechanisms like invasion and suppress the process in both experimental conditions (Figure 6A). A specific explanation could be represented by the presence of Intercellular adhesion molecule 2 (*ICAM2*) in the list of miR-125b target genes (according to data retrieved from mirTargetLink database: https://ccb-web.cs.uni-saarland.de/mirtargetlink/ accessed 15 October 2020, data not presented), molecule that has no specific effect upon colon cancer cell proliferation and viability but is significantly inhibiting would healing in vitro [20]. Silencing of miR-125b can upregulate the expression of *ICAM2* and surpass the effect of other oncogenic genes with role in invasion that are targeted by the same miRNA (e.g., *GLI1,* and *CDH5*). Further studies are necessary to confirm the hypothesis. In HCT-116, the gap closed slightly slower in the case of miR-125b mimic treated cells, but the difference was not significant, and the random effect was not discarded, thus demonstrating an abrogated effect of miR-125b replacement in *TP53* wild-type cells in terms of invasion and migration (Figure 6A).

Due to the significant results obtained in DLD-1, these cells transfected with miR-125b mimic/inhibitor or the controls were cultured to form 3D structures. The spheroids from DLD-1 mimic have a more compact phenotype, as seen by their darker color, in comparison with the control, while for miR-125b inhibitor treated DLD-1 cells, the spheroids have a more invasive aspect, due to their size and loosen aspect, with many gaps between the cells (Figure 6B). When tested in a Matrigel invasive assay, the miR-125b inhibitor stimulated cell invasion by degrading the extracellular matrix, while miR-125b overexpression determined the spheroids to remain compact concomitant with reduced migration through 48 h (Figure 6C).

### 3.5. Targeted Gene Expression Affected by miR-125b Exogenous Expression Modulation

**Figure 7:**
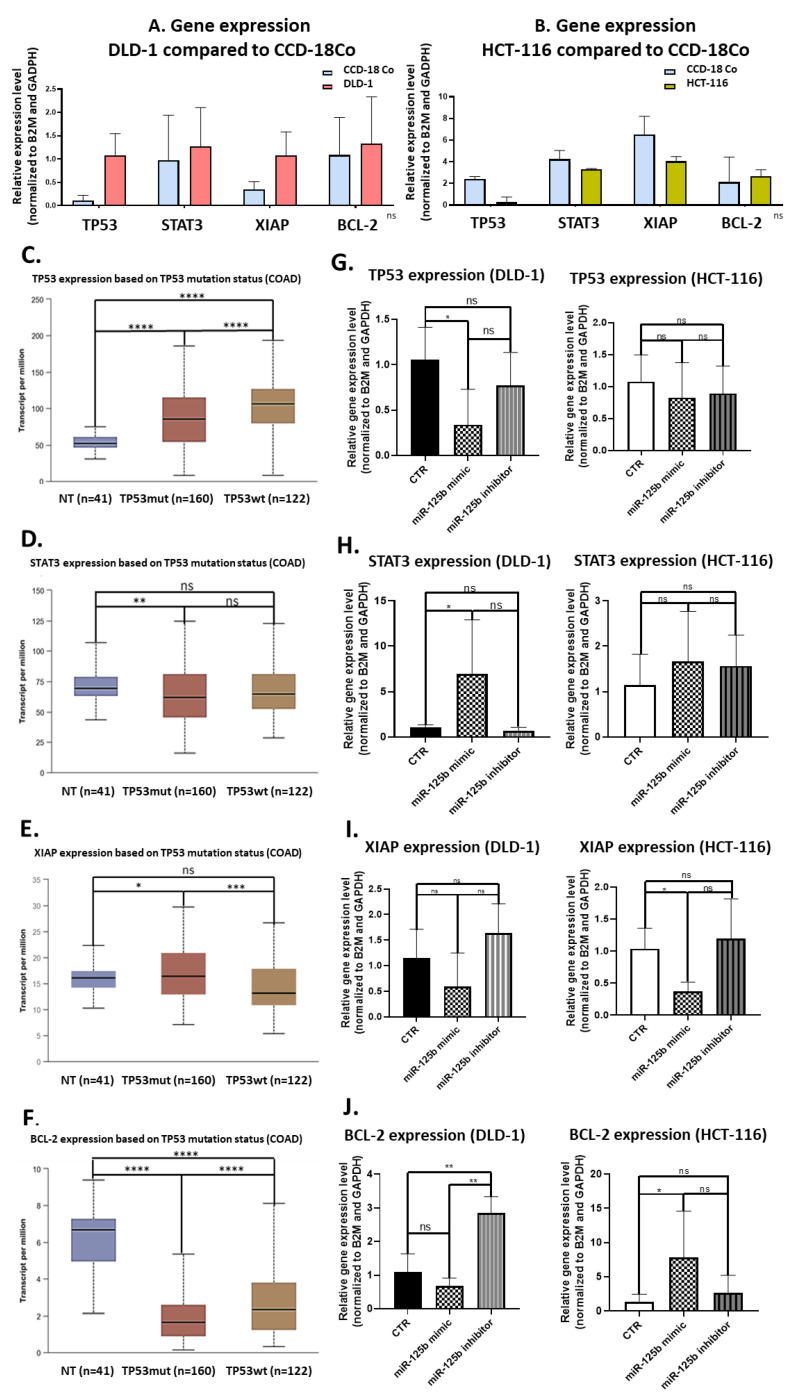
miR-125b-gene interaction network and its changes in colon cancer following miR-125b exogenous modulation. (**A**) RT-qPCR expression of TP53, STAT3, XIAP, and BCL-2 in DLD-1 (colon cancer) compared to CCD-18 cell line (normal) (data presented as mean ± S.D.) (**B**) RT-qPCR expression of TP53, STAT3, XIAP and BCL-2 in HCT-116 (colon cancer) compared to CCD-18 cell line (normal) (data presented as mean ± S.D.) (**C**) TP53 expression in TCGA data of normal adjacent tissue (*n* = 41), colon cancer TP53 mutated tissue (*n* = 160) and colon cancer TP53 wild-type tissue (*n* = 122) (data presented as mean ± S.D., **** *p* < 0.0001 for TP53-Mutant independent *t*-test compared to Normal; **** *p* < 0.0001 for TP53-NonMutant independent *t*-test compared to Normal; **** *p* < 0.0001 for TP53-Mutant independent *t*-test compared to TP53 NonMutant (**D**) STAT3 expression in TCGA data of normal adjacent tissue (*n* = 41), colon cancer TP53 mutated tissue (*n* = 160) and colon cancer TP53 wild-type tissue (*n* = 122) (data presented as mean ± S.D., ** *p* = 0.0079 for TP53 Mutant independent *t*-test compared to Normal) (**E**) XIAP expression in TCGA data of normal adjacent tissue (*n* = 41), colon cancer TP53 mutated tissue (*n* = 160) and colon cancer TP53 wild-type tissue (*n* = 122) (data presented as mean ± S.D., * *p* = 0.010 for TP53 Mutant independent *t*-test compared to Normal, *** *p* = 0.00023 TP53 NonMutant independent *t*-test compared to TP53 Mutant (**F**) BCL-2 expression in TCGA data of normal adjacent tissue (*n* = 41), colon cancer TP53 mutated tissue (*n* = 160) and colon cancer TP53 wild-type tissue (*n* = 122) (data presented as mean ± S.D., **** *p* < 0.0001 for TP53-Mutant independent *t*-test compared to Normal, **** *p* < 0.0001 for TP53-NonMutant independent *t*-test compared to Normal, **** *p* < 0.0001 for TP53-NonMutant independent *t*-test compared to TP53-Mutant) (**G**) RT-qPCR expression of TP53 in DLD-1 and HCT-116 cells following miR-125b mimic (100 nM) or inhibitor (100 nM) transfection, compared to control cells treated with empty Lipofectamine for 48 h (data presented as mean ± S.D.; * *p* = 0.0294 for DLD-1—miR-125b mimic, independent *t*-test compared to Control) (**H**) RT-qPCR expression of STAT3 in DLD-1 and HCT-116 cells following miR-125b mimic (100 nM) or inhibitor (100 nM) transfection, compared to control cells treated with empty Lipofectamine for 48 h (data presented as mean ± S.D.; * *p* = 0.0338 for DLD-1—miR-125b mimic, independent *t*-test compared to Control). (**I**) RT-qPCR expression of XIAP in DLD-1 and HCT-116 cells following miR-125b mimic (100 nM) or inhibitor (100 nM) transfection, compared to control cells treated with empty Lipofectamine for 48 h (data presented as mean ± S.D.; * *p* = 0.0119 for HCT-116—miR-125b mimic, independent *t*-test compared to Control) (**J**) RT-qPCR expression of BCL-2 in DLD-1 and HCT-116 cells following miR-125b mimic (100 nM) or inhibitor (100 nM) transfection, compared to control cells treated with empty Lipofectamine for 48 h (data presented as mean ± S.D.; ** *p* = 0.0022 for DLD-1—miR-125b inhibitor, independent *t*-test compared to Control, ** *p* = 0.0022 for DLD-1—miR-125b inhibitor, independent *t*-test compared to DLD-1—miR-125b mimic, * *p* = 0.0425 for HCT-116—miR-125b mimic, independent *t*-test compared to Control). All RT-qPCR experiments were performed in duplicates for each cell line and experimental protocol and gene relative expression was expressed by fold-change and normalized to B2M and GAPDH.

As previously established, miR-125b is directly targeting *TP53* and B-cell lymphoma 2 (*BCL-2*) with strong experimental support and also Signal transducer and activator of transcription 3 (STAT3) and X-Linked inhibitor of apoptosis (*XIAP*) with weaker experimental support (according to data retrieved from mirTargetLink database: https://ccb-web.cs.uni-saarland.de/mirtargetlink/ accessed on 15 October 2020, data not presented). The expression of these genes was firstly investigated in the two colon cancer cell lines, DLD-1 and HCT-116, in comparison with a normal cell line, CCD-18Co. As presented in Figure 7A,B, in DLD-1, *TP53* is overexpressed (Figure 7A), whereas in HCT-116 is underexpressed (Figure 7B). *STAT3* has similar expression throughout all three cell lines, although slightly elevated in DLD-1 cell line (Figure 7A). *XIAP* is up-regulated in DLD-1 cell line (Figure 7B) and slightly down-regulated in HCT-116 cell line (Figure 7B). *BCL-2* is slightly elevated both colon cancer cell lines (Figure 7A,B). The same genes were also investigated in TCGA colon cancer cohorts (COAD) in dependency to the *TP53* status. As follows, *TP53* is significantly elevated in cancer tissue compared to normal adjacent samples independently of its mutational status, with a slight elevation in the case of the *TP53* wild-type samples compared to both normal samples and *TP53* mutant ones (Figure 7C). *STAT3* expression is significantly downregulated in *TP53* mutant colon cancer tumors, however there is no significant difference in *STAT3* expression between normal tissues and *TP53* wild-type colon cancer tumors and between tumor tissue with the two types of *TP53* status (Figure 7D). For the case of *XIAP* gene, its expression is higher in *TP53* mutant colon cancer samples versus normal tissue (Figure 7E). In *TP53* wild-type tissue, there is no statistically significant change in expression of *XIAP* compared to normal tissue, however, its expression is significantly down-regulated, when compared to *TP53* mutant tumor tissue (*p* = 0.00023538) (Figure 7E). *BCL-2* is strongly downregulated in colon cancer tissues, independent of *TP53* mutational status with a more accentuated loss in *TP53* mutant tissue samples (Figure 7F).

Modulation of miR-125b expression in colon cancer cells, DLD-1 and HCT-116, was followed by changes in the target genes expression as shown in Figure 7. Specifically, *TP53* is significantly inhibited upon miR-125b replacement therapy in *TP53* mutant colon cancer cells (DLD-1) compared to control (Figure 7G), followed by an overexpression of *STAT3* for the same experimental protocol (Figure 7H). No significant changes were present in the case of miR-125b inhibition (most probably due to an already very low level of miR-125b in parent cells). These data demonstrate a novel interconnection between mutant *TP53* partial loss of function and *STAT3* activation, similar to a feedback mechanism. Moreover, it seems that *STAT3* is not targeted by miR-125b in DLD-1 cells. For the case of HCT-116, miR-125b mimic did not significantly affect the level of *TP53*, meaning that in the case of *TP53* wild-type cells this gene is not prioritized by the miRNA in targeting activities (Figure 7G). In response to the slight downregulation of TP53 following miR-125b replacement, the same feedback mechanisms represented by *STAT3* upregulation is recognized, but without statistical significance (Figure 7H). *XIAP* seems like a true target of miR-125b, being downregulated in both cell types after miR-125b addition and slightly upregulated upon miRNA inhibition (Figure 7I). The same gene was previously associated with promotion of colony formation and proliferation in colon cancer [21], both mechanisms abrogated in miR-125b treated colon cells, especially in the *TP53* mutant cells. An opposite effect of miR-125b mimic was observed in the case of *BCL-2* gene that was identified as downregulated in DLD-1 cells (according to the status of direct target) but upregulated in HCT-116 (Figure 7J). A significant upregulation was observed in the moment of miR-125b inhibition in the case of DLD-1 (Figure 7J). However, according to a recent meta-analysis [22], high expression of *BCL-2* is associated with a good prognosis in colorectal cancer with better overall survival and disease-free survival rates. Even so, the upregulation of *BCL-2* did not cause significant phenotypical changes in HCT-116 cells as demonstrated by the functional studies.

Overall, according to the gene expression data it seems that the difference in response to miR-125b replacement therapy between the two colon cancer cells is mainly caused by the differential modulation of *TP53* expression and implicitly its mutational status. Even so, an array type of study is further necessary to confirm the therapeutic role of miR-125b only in patients with functional *TP53* mutations.

## 4. Discussion

miR-125b has a controversial role in malignant disease, with significant differences among different cancer types, depending on the targeted genes and their role in a particular cancer development. However, specific reasons behind these contrasting roles are yet to be identified [12]. In bladder cancer, miR-125b was reported to be generally down-regulated, where overexpression of this miRNA inhibits the capacity of cancer cells to form colonies or xenografts in immunocompromised mice [23]. Moreover, through regulation of Matrix metalloproteinase 13 (*MMP13*), miR-125b is also mediating the invasion and metastasis process [24]. The downregulation of miR-125b was also confirmed in the local cohort of bladder cancer tissue samples versus normal adjacent ones. For the case of breast cancer, the promoter region of miR-125b gene is methylated; thus, this miRNA is also generally reported as under expressed in the reminded pathology [25]. In this case the function of miR-125b is especially related to cellular proliferation, by indirect downregulation of Ras-mitogen activated protein kinase (*MAPK*) and inhibition of apoptosis through mammalian target of rapamycin (*mTOR*) pathway [12,26,27]. In our local cohort of double positive breast cancer tissue, as well as in our analysis of TCGA RNASeq data, the literature data was confirmed.

In colorectal cancer, reports of miR-125b upregulation are associated with poor prognosis, but without an identified mechanism [12]. An analysis of 89 colorectal cancer cases concluded that miR-125b upregulation is associated with advanced tumor stage and invasion of local tissue. The role of miR-125b was linked to inhibition of the tumor suppressor gene, *TP53* [13], but without considering the possible mutational alterations that can intervene within these gene in colorectal malignancies. In our analysis, miR-125b was down-regulated in colon cancer tissue samples in both the local cohort and TCGA RNASeq data. A recently published study reported miR-125 down-regulation in colon cancer cell lines and proved that the activity of this miRNA is related to impaired apoptosis and invasion, through *TAZ* gene targeting [14]. miR-125b is also down-regulated in colon cancer chemoresistant cell lines, as a result of Toll-like receptor (*TLR*) 2/6 and *TRL5* activation. miR-125b mimic re-sensitized the cells and reversed the overactivation of the invasion process [28]. Moreover, miR-125b hypermethylation has also been reported in colon cancer, process generally associated with decreased expression [29].

For the present study, in connection to the previous ones, and also to the fact that the function of *TP53* (target of miR-125b) in cancer is highly dependent on its mutational status, the decision was made to test whether miR-125b role in colon cancer is dependent on the mutation status of *TP53*. miR-125b is downregulated in both colon cancer cell lines (DLD-1 and HCT-116) and tissue samples, data confirmed by TCGA RNASeq values for COAD cohort. Moreover, this miRNA is targeting *TP53* that is upregulated in COAD samples from TCGA database. *TP53* is highly expressed in DLD-1 cell line that carries high mutation burden including an exonic mutation in *TP53* gene and is also associated with a more aggressive phenotype in comparison with HCT-116 colon cell line that is *TP53* wild-type; the expression of *TP53* in HCT-116 was found at low levels compared to the normal cell line. miR-125b mimic or inhibitor transfection in both colon cancer cell line did not significantly affect the percentage of apoptotic/necrotic cells, or cell cycle progression. However, the mimic transfected cells have a lower confluence and increased morphological stress in the cytoskeleton architecture. The therapeutic role of miR-125b replacement in DLD-1 cell line was observed in the case of impaired colony forming capacity and invasion and ability to form spheroid structures; in the case of the inhibitor the malignant potential of the *TP53* mutated cells was enhanced. Addition of miR-125b in HCT-116 cell line did not impose a significant effect upon these hallmarks. These results prove that miR-125b has a tumor suppressor role in *TP53* mutated colon cancer cells, while it does not significantly affect the behavior of *TP53* wild-type cells.

At molecular level, increased levels of miR-125b determined a significant suppression of *TP53* in DLD-1 cells, while minimal changes were observed in HCT-116 compared to control samples. The downregulation of *TP53* was further associated with an contradictory increase in *STAT3* expression, gene that is also a target of miR-125b, demonstrating a possible novel inter- dependency between *TP53* and *STAT3* and also a non-direct modulation of *STAT3* by miR-125b (although *STAT3* is confirmed as a target of miR-125b). This result is favoring the malignant development, considering that *STAT3* plays an oncogenic role in colon cancer [30]. *XIAP* gene, a direct target of miR-125b was found as inhibited after miR-125b replacement and upregulated within miR-125b inhibitor treated cells. A similar profile was found in both colon cancer cells, confirming the role of miR-125b as a direct modulator of *XIAP* and implicitly an inhibitor of cancer growth considering the oncogenic role of *XIAP* gene in colon malignancies [21]. miR-125b causes *BCL-2* down-regulation in miR-125b mimic treated DLD-1 cells and up-regulation in miR-125b inhibitor treated DLD-1 cells. What is further interesting is that *BCL-2* was overexpressed in HCT-116 transfected with miR-125b mimic. *BCL-2* overexpression is considered a favorable prognostic factor in colon cancer [31], especially if associated with wild-type *TP53* status [32].

In conclusion, our results show the importance of differentiating between *TP53* mutation status when assessing the role of miR-125b in colon cancer, and not only. The in vitro results showed that in colon cancer cell lines, mutated *TP53* is inhibited by exogenous miR-125b causing inhibition of colony formation ability, invasion, and metastasis, while wild-type *TP53* is not affected, concomitant with minimal phenotypical differences. Further research, on clinical samples and in vivo experiments are needed to investigate the dual role of miR-125b-*TP53* in colon and colorectal cancer.

## 5. Future Perspectives

The present study, besides the investigation of miR-125b therapeutic activity in colon cancer, is standing as an example regarding the importance of the mutational status of miRNA targeted genes taken toward investigation. Moreover, while a miRNA is downregulated in a specific malignancy, is not necessarily a true tumor suppressor or is not associated with a complete tumor suppressor role. As an example, miR-125b is reducing the expression of mutated *TP53* and *XIAP* (oncogenic in colon cancer), while enhancing the level of *STAT3* (oncogenic in colon cancer) and inhibiting the expression of *BCL-2* (tumor suppressor in colon cancer) in DLD-1 cells. Moreover, in a different cellular scenario (HCT-116), miR-125b is acting mainly as a tumor suppressor by reducing the levels of *XIAP* and positively modulating the expression of *BCL-2,* but without a visible phenotypical effect (probably due to additional modulation on counteracting genes). Therefore, in our opinion, miRNAs should be used only as sensitizers of the cancer cells through disbalance of the signaling profile prior to anti-cancer drugs and not as true therapeutic agents [6].

## Supplementary Materials

The following are available online at https://www.mdpi.com/article/10.3390/pharmaceutics13050664/s1, Figure S1: miR-125b expression in breast and bladder cancer from TCGA data and from local cohorts. **A.** RT-qPCR results of miR-125b-5p expression in double positive breast cancer tissue samples (*n* = 44) versus normal adjacent tissue samples (*n* = 21) (data presented as mean ± S.D.; ** *p* = 0.0014, two-tailed Mann Whitney test) **B**. RT-qPCR results of miR-125b-5p expression in bladder cancer tissue samples (*n* = 37) versus normal adjacent tissue samples (*n* = 39) (data presented as mean ± S.D.; *** *p* = 0.0003, two-tailed Mann Whitney test). Experiments including the local cohorts of patients were performed in duplicate for each sample and miR-125b-5p relative expression was expressed by fold-change and normalized to U6 snRNA **C.** Validation of miR-125b-5p expression (RNASeq count) on BRCA cohort from TCGA database in tumor tissue (*n* = 1098) compared to normal adjacent ones (*n* = 104) (data presented as mean ± S.D.; **** *p* < 0.0001, two-tailed Mann Whitney test). **D.** Validation of miR-125b-5p expression (RNASeq count) on BLCA cohort from TCGA database in tumor tissue (*n* = 413) compared to normal adjacent one (*n* = 19) (data presented as mean ± S.D.; **** *p* < 0.0001, two-tailed Mann Whitney test).
