# Supplementary material for: Hsa-miR-125b Therapeutic Role in Colon Cancer Is Dependent on the Mutation Status of the TP53 Gene
Source: Pharmaceutics. 2021 May 6;13(5):664. doi: 10.3390/pharmaceutics13050664 (PMC8148199; doi:10.3390/pharmaceutics13050664)
Supplement: Supplementary file 1 [file pharmaceutics-13-00664-s001.zip › pharmaceutics-1191973-supplementary.pdf]

pharmaceutics

MDPI

Supplementary Materials: Hsa-miR-125b Therapeutic Role in Colon Cancer Is Dependent on the Mutation Status of the TP53 Gene

Diana Cenariu, Alina-Andreea Zimta, Raluca Munteanu, Anca Onaciu, Cristian Moldovan, Ancuta Jurj, Lajos Raduly, Alin Moldovan, Adrian Florea, Liviuta Budisan, Laura-Ancuta Pop, Lorand Magdo, Mihai Albu, Rares Tonea, Mihai-Stefan Muresan, Calin Ionescu, Bogdan Petrut, Rares Buiga, Alexandru Irimie, Diana Gulei and Ioana Berindan-Neagoe

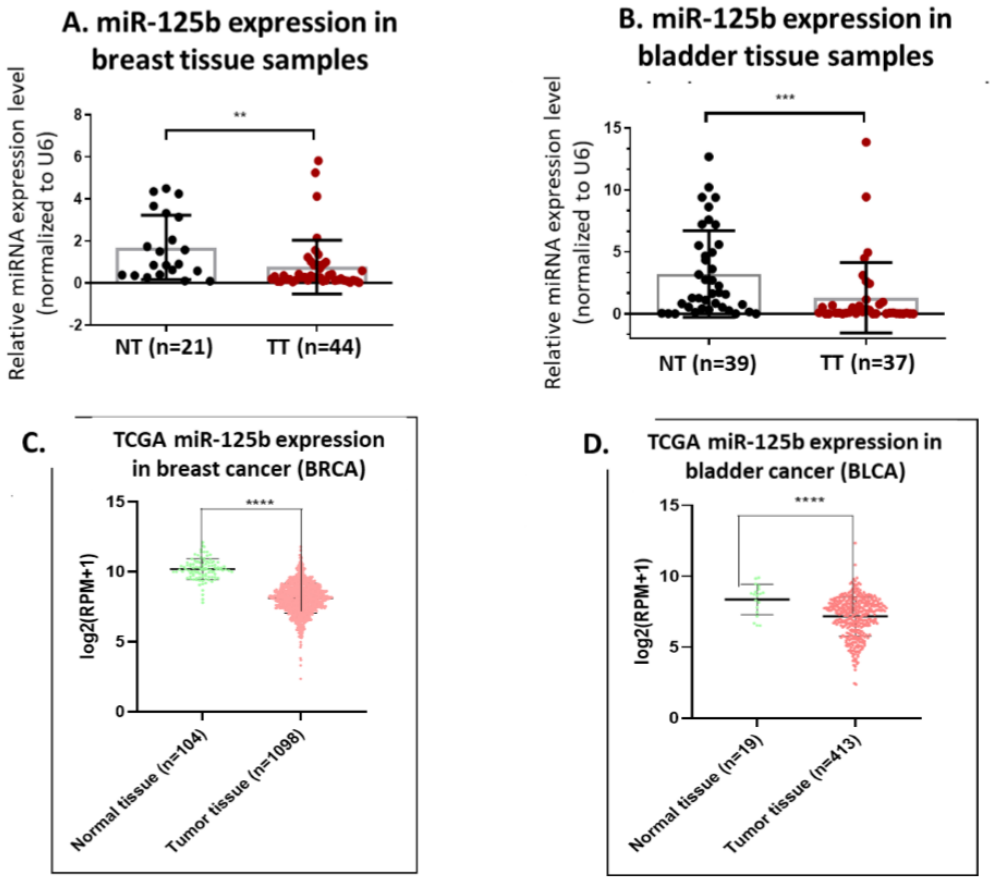

Figure S1. Figure S1: miR-125b expression in breast and bladder cancer from TCGA data and from local cohorts. **A.** RT-qPCR results of miR-125b-5p expression in double positive breast cancer tissue samples (*n*=44) versus normal adjacent tissue samples (*n*=21) (data presented as mean ± S.D.; **$p = 0.0014$, two-tailed Mann Whitney test) **B**. RT-qPCR results of miR-125b-5p expression in bladder cancer tissue samples (*n*=37) versus normal adjacent tissue samples (*n*=39) (data presented as mean ± S.D.; ***$p = 0.0003$, two-tailed Mann Whitney test). Experiments including the local cohorts of patients were performed in duplicate for each sample and miR-125b-5p relative expression was expressed by fold-change and normalized to U6 snRNA **C.** Validation of miR-125b-5p expression (RNASeq count) on BRCA cohort from TCGA database in tumor tissue (*n*=1098) compared to normal adjacent ones (*n*=104) (data presented as mean ± S.D.; ****$p < 0.0001$, two-tailed Mann Whitney test). **D.** Validation of miR-125b-5p expression (RNASeq count) on BLCA cohort from TCGA database in tumor tissue (*n*=413) compared to normal adjacent one (*n*=19) (data presented as mean ± S.D.; ****$p < 0.0001$, two-tailed Mann Whitney test).
